# Generating enhanced mucosal immunity against *Bordetella pertussis:* current challenges and new directions

**DOI:** 10.3389/fimmu.2023.1126107

**Published:** 2023-02-21

**Authors:** Amanda D. Caulfield, Maiya Callender, Eric T. Harvill

**Affiliations:** Department of Infectious Diseases, College of Veterinary Medicine, University of Georgia, Athens, GA, United States

**Keywords:** pertussis, whooping cough, mucosal immunity, sterilizing immunity, animal models, waning immunity, pulmonary disease

## Abstract

*Bordetella pertussis (Bp)* is the highly transmissible etiologic agent of pertussis, a severe respiratory disease that causes particularly high morbidity and mortality in infants and young children. Commonly known as “whooping cough,” pertussis is one of the least controlled vaccine-preventable diseases worldwide with several countries experiencing recent periods of resurgence despite broad immunization coverage. While current acellular vaccines prevent severe disease in most cases, the immunity they confer wanes rapidly and does not prevent sub clinical infection or transmission of the bacterium to new and vulnerable hosts. The recent resurgence has prompted new efforts to generate robust immunity to *Bp* in the upper respiratory mucosa, from which colonization and transmission originate. Problematically, these initiatives have been partially hindered by research limitations in both human and animal models as well as potent immunomodulation by *Bp.* Here, we consider our incomplete understanding of the complex host-pathogen dynamics occurring in the upper airway to propose new directions and methods that may address critical gaps in research. We also consider recent evidence that supports the development of novel vaccines specifically designed to generate robust mucosal immune responses capable of limiting upper respiratory colonization to finally halt the ongoing circulation of *Bordetella pertussis.*

## Introduction

*Bordetella pertussis (Bp)* is the highly transmissible etiologic agent of pertussis, a severe respiratory disease that causes particularly high morbidity and mortality in infants and young children (1–3). Patients with pertussis disease classically exhibit bouts of intense paroxysmal coughing which may be accompanied by post-tussive vomiting, bronchopneumonia, pulmonary hypertension, hypoxia, and in severe cases, brain damage or death (4). Problematically, whooping cough remains one of the least controlled vaccine-preventable diseases worldwide with several countries experiencing recent periods of resurgence (5). Increased incidence has been correlated with a transition from whole cell pertussis (wP) vaccines to acellular pertussis (aP) vaccines composed of 1-5 detoxified antigens including pertussis toxin, pertactin, filamentous hemagglutinin, and fimbriae 2&3 (6–9). While aP vaccines are highly successful in preventing severe lower respiratory (LR) disease in most cases, the Th-2 skewed immunity they confer wanes rapidly and fails to prevent sub-clinical upper respiratory (UR) mucosal infection and transmission to new hosts (9, 10).

These observations are compounded by the rapid emergence and expansion of “vaccine escape mutant” strains deficient in pertactin, the only antigen included in aP vaccines capable of generating bactericidal antibodies against *Bp* (7, 8, 11–14). It is speculated that targeted loss of this surface-bound antigen, but not other aP components, may grant a fitness advantage as it evades anti-PRN bactericidal antibodies to facilitate persistence within the UR tract of aP vaccinated hosts (8). This is further supported by the observation that pertactin-deficient mutants are emerging from a diversity of *Bp* lineages, which have now risen to dominance in many countries with broad aP vaccine coverage. Together these observations have prompted new research initiatives aimed at generating sterilizing mucosal immunity in the nasal cavity to prevent initial colonization and limit the ongoing circulation of *Bp* within vaccinated populations.

Of note, the issue of sub-clinical infection and transmission amongst vaccinated individuals is not limited to *Bp.* Generating sterilizing immunity in the nasopharynx is a common challenge against many respiratory pathogens including pandemic SARS-CoV-2, *Mycobacterium tuberculosis*, influenza, respiratory syncytial virus, and others (15–18). Each of these pathogens can infect, persist, and often transmit from the nasopharynx; a mucosal site that continuously encounters foreign antigen. To do this, these pathogens must bypass specialized nasopharyngeal tissue equipped with mucus layers saturated with antimicrobial peptides, beating cilia, a sea of competitive microflora, innate responders such as neutrophils & macrophages, as well as inductor and effector sites replete with dendritic cells, T cells, B cells, and secretory IgA (19–22).

In humans, these complex defensive networks are largely evaded or modulated by *Bp* long enough to facilitate growth and transmission to new hosts (19, 23–30). Increased reports of asymptomatic or sub-clinical cases of *Bp* infection provides clinical evidence of this ability in populations with broad vaccine coverage which warrants new research initiatives and the development of targeted mucosal vaccine strategies. To develop vaccines capable of generating sustained protective immunity in the UR mucosa, it is critical that we first understand how these pathogens modulate or evade the host immune response in the nasopharynx and similarly determine which mucosal immune factors are involved in eventually controlling and clearing infection.

Much of our understanding of host-pathogen interactions in *Bp* infections has been derived from conventional animal models aimed at characterizing (and preventing) the most severe forms of pertussis disease (19). These efforts have been instrumental in understanding the systemic and pulmonary immune responses to *Bp*, enabling the development of vaccines capable of preventing severe life-threatening pertussis. However, despite *Bp* primarily residing within the upper respiratory mucosa, there is comparatively little published work that investigates the specific local mucosal immune response to *Bp* relative to pulmonary or systemic responses (31). Excitingly, the recent development of several novel murine and baboon models has begun to allow us to study the critical mechanistic distinctions between UR and LR responses, and elucidate factors involved in colonization, persistence, transmission, and immunity in *Bp* infections. Although much more arduous and expensive, controlled human infection models have the potential to verify results observed in precursory animal models and expand our understanding of the human-specific response to *Bp*.

Here we consider our incomplete understanding of the complex host-pathogen dynamics occurring in the upper airway to highlight and propose new directions that may address these critical gaps in research. We also consider recent evidence that supports the development of intranasal vaccines specifically designed to generate sustained mucosal immune responses capable of limiting upper respiratory colonization and the ongoing circulation of *Bordetella pertussis.*

## Intramuscular vaccine-induced serum IgG protects against severe pulmonary disease but does not control *Bp* in the nasal cavity

While the cause of pertussis resurgence is multifactorial, it is well-appreciated that waning immunity and flawed aP vaccine-induced immunity are involved. Problematically, vaccine-induced protection and antibody titers roughly correlate, but neither is sustained indefinitely. Prior data from several human studies have demonstrated that peripheral blood anti-pertussis immunoglobulin G (IgG) titers decay rapidly within the first-year post-vaccination and continue to decline at slower rates thereafter (32–37). However, it remains unclear at what threshold antibody-titer decay results in increased susceptibility to pertussis disease. Although it is currently believed that high anti-pertussis IgG titers contribute to prevention of severe pulmonary disease, the mechanisms of protection remain poorly understood, and appear to vary in different regions of the respiratory tract (10, 19, 22, 38–41).

In contrast to protection observed in the LR tract, vaccine-induced IgG responses are less effective in controlling UR mucosal *Bp* infection. This has been repeatedly observed from conventional murine models, as high antibody titers were detected in aP vaccinated animals with no reduction in CFU in the nasal cavity, resulting in prolonged carriage (19, 21). This was similarly reported in the aP-primed baboon model, which demonstrated persistent colonization beyond 35-days post-challenge despite high serum IgG titers against all five aP antigens. Interestingly, the lack of IgG-mediated protection is not unique to aP vaccine-induced immunity and was also reported from wP primed baboons with modest improvement in clearance to 19/21 days post-challenge (10). Together these observations suggest aP- and wP-primed IgG responses fail to rapidly control *Bp* from the nasopharynx, resulting in extended periods of colonization and transmissibility.

Historically, anti-pertussis IgG titers have been the most accessible indicator for immunity in both clinical and research settings. However, the dual observation that both wP and aP vaccine-induced IgG titers correlate with reduced incidence of severe pulmonary disease *but fail* to prevent sub-clinical infection complicates our ability to use anti-pertussis IgG as a reliable indicator for UR protection. The disconnect between high IgG titers and persistent nasopharyngeal infection may be partially attributed to pertussis toxin-specific mechanisms that block serum-mediated clearance by delaying the recruitment of neutrophils, among other immunomodulatory abilities detailed in sections below (26). These data collectively suggest that serum IgG titers alone are not a sufficient indicator for protective immunity against *Bp*, and that other, localized mechanisms of protection should be evaluated.

It is well appreciated that convalescent immunity following a course of primary infection with *Bp* generates humoral *and* cellular immune responses that are critical in generating more effective protection against secondary infection in both the UR and LR tracts (31). It is probable that a *portion* of this protective immune response in the LR is mediated by IgG, and evidence supporting its role in reducing morbidity and mortality associated with clinical disease warrants continued research into anti-pertussis IgG responses. However, when evaluating IgG responses to new candidate antigens for improved vaccines, it is critical that we differentiate between bactericidal antibodies directed against surface-bound factors which facilitate clearance, and neutralizing antibodies which protect against toxin-induced severe disease (11).

## The controversial role of secretory IgA against *Bp*

Immunoglobulin A (IgA) is produced in response to natural infection, but not from current intramuscular aP or wP vaccines. However, conflicting reports regarding the role of IgA in infection with *Bp* have confounded our ability to correlate protection with this critical component of mucosal immunity. Anti-pertussis IgA appears to reduce adherence of *Bp* to ciliated epithelium *in vitro*, which could provide some level of protection against initial colonization and/or spread through the respiratory tract (39). In addition, human anti-pertussis IgA has been shown to effectively bind FcαRI on polymorphonuclear leukocytes to stimulate phagocytosis *in vitro*, indicating that IgA can be effective in facilitating bacterial clearance, despite IgA being traditionally considered a poor complement activator relative to IgM and IgG (22, 42). However, IgA-deficient mice were previously reported to be indistinguishable from wild type mice in controlling primary or secondary *Bp* infection, suggesting IgA plays at most a modest and/or redundant role in immunity to *Bp* (41).

The strongest data to argue a protective role for IgA against *Bp* was reported from experiments investigating a recent live-attenuated vaccine candidate, BPZE1. Designed to be administered intranasally, BPZE1 is reported to generate a robust secretory IgA response which appears to correlate with protection against challenge with *Bp.* Importantly, BPZE1-vaccinated IgA-/- mice had significantly higher colonization on days 7 and 21 post-challenge relative to vaccinated wildtype mice (43). These data indicate that removal of IgA resulted in significantly impaired protection generated by a candidate intranasal vaccine. Importantly, baboons vaccinated with BPZE1 also generated protective immunity with detectable *Bp-*specific serum IgA, however UR mucosal sIgA was not directly evaluated (44).

The observation of sIgA production following *intranasal* delivery of commercial pertussis vaccines has prompted new investigations into the merits of intranasal *vs.* intramuscular vaccination approaches. In a recent study, mice intranasally vaccinated with aP vaccines generated similar levels of protection and serum IgG levels relative to intramuscular vaccination *and* significantly higher IgA titers in the nasal cavity by day 9 post *Bp* challenge (45, 46). A small human study also observed strong mucosal *and* systemic immune responses following intranasal wP vaccination (over 4 doses), which is a positive indicator for applicability of this concept in human immunization (47).

**Figure 1:**
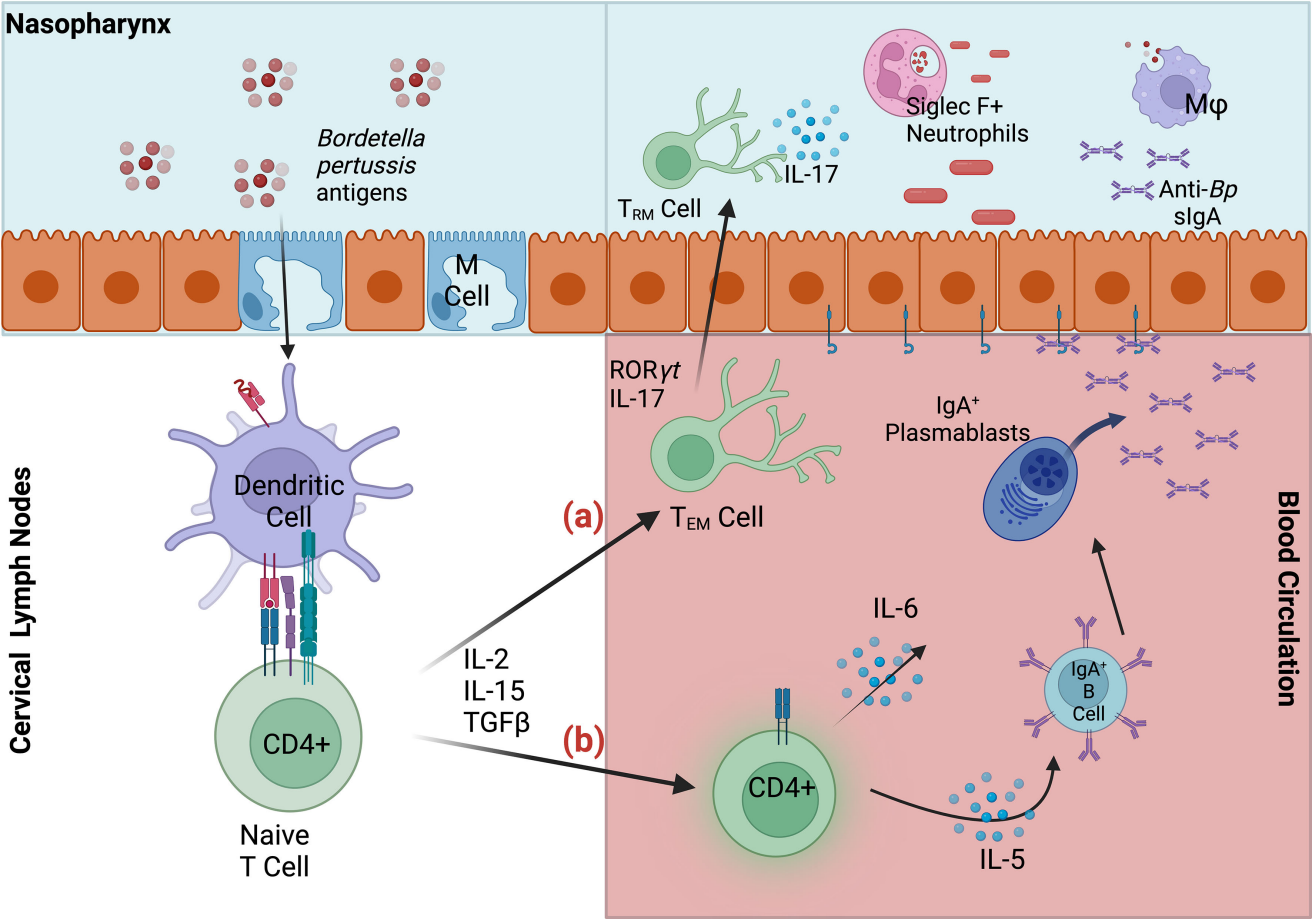
Summary of mucosal responses to *Bordetella pertussis* in nasopharynx-associated lymphoid tissue (NALT). *Bp* antigens are transported *via* microfold **(M)** cells into the NALT, where dendritic cells capture antigen, migrate to the lymph nodes and stimulate naïve CD4^+^ T cells. **(A)** Activated effector CD4^+^ T cells may then migrate to nasal tissue, where they are maintained as T_RM_. Upon secondary infection IL-17^+^ T_RM_ expand and recruit Siglec F^+^ neutrophils to the nasopharynx, facilitating rapid clearance **(B)** Effector CD4^+^ T cells also induce maturation of IgA-committed B cells, which migrate to the cervical lymph nodes, and to their effector site (nasal cavity). IgA^+^ B cells and plasmablasts mature into plasma cells in response to cytokines IL-5 and IL-6. Dimeric IgA, secreted by differentiated plasma cells, bind polymeric Ig receptors, and are released into infected respiratory tissue as secretory IgA. Anti-*Bp* sIgA, along with macrophages (Mφ) and Siglec F+ neutrophils contribute to the clearance of *Bp* in the upper respiratory tract.

These examples suggest that intranasal vaccination and/or infection are able to generate protective sIgA against *Bp via* a site-specific mechanism. It is likely that intranasal but not intramuscular delivery facilitates uptake by nasal microfold (M) cells required for translocation into nasopharynx-associate lymphoid tissue (NALT) (48). This inductive tissue is critical for the generation of mucosal immunity against other pathogens, and NALT-targeted immunization has been shown to induce *both* mucosal and systemic immunity. Importantly, sIgA production is specifically stimulated in mucosal effector sites, where dendritic cell-activated T-cells specifically induce clonal expansion of IgA^+^ B cells (**Figure 1**). The resulting IgA^+^ B cells and plasmablasts induced by nasal immunization express CCR10 and α_4_β_1_-integrin, which together facilitate migration to respiratory tissues that express the corresponding receptors CCL28 and VCAM1, respectively (48–50).

In sum, conflicting reports of IgA-mediated protection against *Bp* warrant additional investigations into sIgA and intranasal vaccination strategies that deliver antigen directly to the site of infection (50). However, linking sIgA responses with *sustained* protection may present an additional challenge for future vaccine applications aimed at eliciting this response given the faster decline of IgA relative to IgG titers following diagnosis of clinical (symptomatic) pertussis (42, 51).

## New vaccines must generate robust Th1/Th17 responses to enable clearance of *Bordetella pertussis* from the nasopharynx

The inability of aP vaccines to generate robust immunity in the nasal mucosa has sparked a critical need to better understand the immune response to *Bp* at this initial site of infection, in contrast to pneumonic responses which are relatively well-characterized. To date, cellular responses specific to *Bp* in the nasal mucosa have received the most attention, however, research initiatives investigating this topic are relatively recent, and much remains unknown.

T cell-mediated immunity has long been reported to play a central role in the control and clearance of *Bp* from the respiratory tract. Specifically, proinflammatory Th1 and Th17 responses have been shown to be critical for protection against colonization with *Bp* (50, 52). Problematically, aP vaccines primarily induce a Th2-skewed response, which is not sufficient to protect the UR tract from subsequent infection (31, 50, 52). Additionally, aP vaccines fail to generate tissue-resident memory T cells (T_RM_), which are typically maintained in respiratory tissue and respond rapidly to secondary encounter with *Bp* (53).

**Figure 2:**
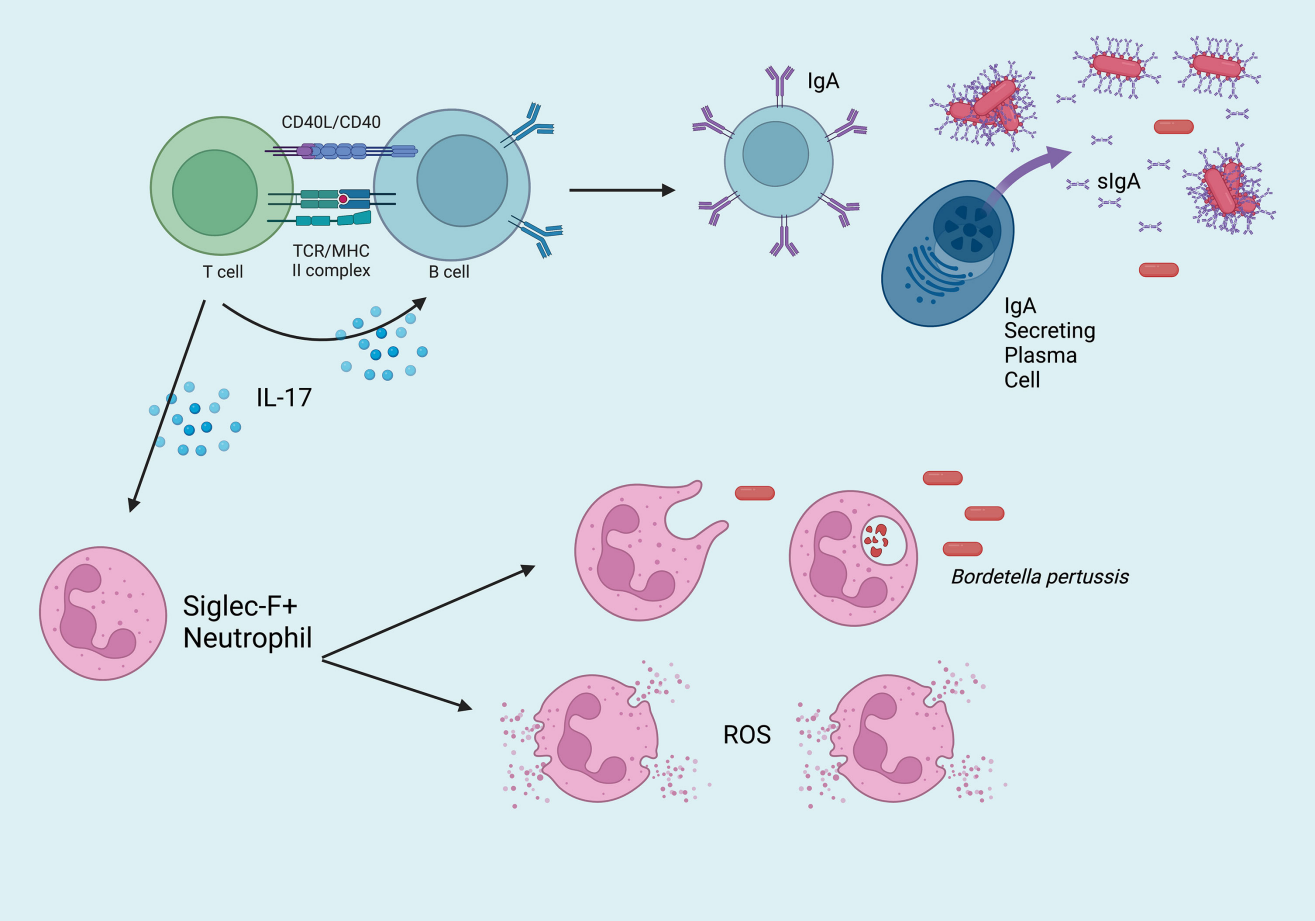
Overview of the critical mucosal-specific factors in cell-mediated immunity to *Bordetella pertussis* in the nasopharynx. Effector CD4^+^ T cells activate IgA^+^ B cells, which mature into dimeric IgA-secreting plasma cells. Once transported across the epithelium, sIgA binds *Bp*, which may bind and enable clearance by phagocytes. IL-17^+^ T_RM_ recruit Siglec F^+^ neutrophils to the site of infection, which eliminate *Bp via* phagocytosis or NETosis (ROS = reactive oxygen species).

Natural infection and intranasal vaccination generate T_RM_ that are protective in the upper respiratory tract (**Figure 1**). Specifically, IL-17 secreting CD4^+^ T_RM_ induce rapid neutrophilic recruitment to the nasopharynx in response to secondary challenge, which has been shown to greatly contribute to the opsonization and control of *Bp* (52–54). Following secondary infection, a cascade of pro-inflammatory cytokines including IL-1β, TNFα, IL-17A (from T_RM_), IL-17C (from epithelial cells), IL-6, and IFNγ recruit additional phagocytes to the site of infection including dendritic cells and macrophages which ultimately clear the infection *via* opsonization-enhanced phagocytosis (**Figure 2**). Interestingly, IL-17^+^ T_RM_ have been shown to recruit a unique subset of Siglec F^+^ neutrophils, which have been recently described to primarily reside within the nasal mucosa and exhibit an activated phenotype with increased NETosis (55, 56).

Decades of immunology research has demonstrated that the absence or removal of a single component of the immune system can result in deleterious effects to protective immunity. Notably, intramuscular aP vaccines fail to stimulate or generate multiple components of both humoral (IgA) and cellular (IL-17^+^ T_RM_, Siglec F^+^ neutrophils) immunity that are critical in the mucosal response to respiratory pathogens. However, the observation that intranasal administration of aP vaccines and candidate vaccines like BPZE1 are able to generate these responses suggest we have much to learn from continued investigations in this area (31).

## Disrupting immunomodulation to inform vaccine-development

An additional barrier to our understanding of the response to *Bp* in the nasopharynx is immunomodulation. Like many well-adapted pathogens, *Bp* has evolved complex mechanisms to modulate the host immune system to its advantage in active infection and can reinfect convalescent hosts after immunity wanes. These abilities are well-accepted to be multifactorial, with several known virulence factors having unique impacts on diverse aspects of the host immune response to include the suppression of neutrophilic inflammation, decreased cytokine signaling from multiple cell types, complement evasion, and suppression of serum antibody responses (23–30).

To highlight a few examples: pertactin has been reported to modulate the secretion of pro-inflammatory cytokines (TNFα, IL-6, IL-8, G-CSF) and may downregulate host genes associated with cell death (30). Adenylate cyclase toxin targets Cd11b-positive professional phagocytes, including dendritic cells, macrophages, and neutrophils by forming cation-selective pores to permeabilize the cell membrane, while also impairing dendritic cell maturation and cytokine secretion (25). Pertussis toxin disrupts G protein-coupled receptor signaling and inhibits the early recruitment of macrophages and neutrophils (24, 26). *B. pertussis* also evades phagocyte-mediated killing by surviving within macrophages, an ability that is shared by many other bacteria with immunomodulatory abilities (57).

Future vaccine candidates, particularly live-attenuated, may benefit from removal of these immunomodulatory factors to boost immunity. Their formulations could be supplemented with detoxified versions of these proteins if their antigens are critical in *Bp* immunity, such as pertussis toxin. It is likely that immunomodulation is particularly important in mucosal immunity to *Bp*, as the tightly controlled inflammatory responses in the nasopharynx may be further reduced to enable persistence and extend the window of transmissibility (19, 58–61).

Disrupting immunomodulation in animal models may be informative as a tool to evaluate which aspects of the host response are actively suppressed by *Bp* (62). This idea is supported by recent work published in animal models of *Bordetella bronchiseptica (Bb)* infection, where deletion of a regulator of immunomodulators (*btrS*) generated robust sterilizing immunity *and* identified eosinophils as unexpected contributors in pulmonary responses to *Bb* (63).

## Concluding remarks

In an era of widespread resurgence, imperfect waning immunity, and rapid expansion of vaccine-escape mutants, research initiatives have ambitiously shifted toward generating complete protection in the nasal mucosa. Accomplishing this goal will require an improved understanding of the complex immunobiology of upper respiratory infection with *Bp*, and the development of vaccines that prime IgG, IgA, and Th1/Th-17 responses in the respiratory mucosa. Excitingly, recent investigations into intranasal vaccination support our ability to develop next-generation vaccines to generate robust sterilizing mucosal and systemic immunity sufficient to reduce carriage and the ongoing circulation of *Bp* in vaccinated populations.

## Data availability statement

The original contributions presented in the study are included in the article/supplementary material. Further inquiries can be directed to the corresponding author.

## Author contributions

AC and MC conceived the perspective. AC and EH wrote the manuscript. MC generated the figures. All authors contributed to the article and approved the submitted version.
